# High sedentary behavior and low physical activity among adults in Afghanistan: results from a national cross-sectional survey

**DOI:** 10.3389/fpubh.2023.1248639

**Published:** 2023-09-19

**Authors:** Supa Pengpid, Ahmad Siyar Noormal, Karl Peltzer

**Affiliations:** ^1^Department of Health Education and Behavioral Sciences, Faculty of Public Health, Mahidol University, Bangkok, Thailand; ^2^Department of Public Health, Sefako Makgatho Health Sciences University, Pretoria, South Africa; ^3^Department of Healthcare Administration, College of Medical and Health Science, Asia University, Taichung, Taiwan; ^4^Heidelberg Institute of Global Health, Heidelberg University, Heidelberg, Germany; ^5^Department of Psychology, University of the Free State, Bloemfontein, South Africa; ^6^Department of Psychology, College of Medical and Health Science, Asia University, Taichung, Taiwan

**Keywords:** sedentary behavior, low physical activity, adults, Afghanistan, STEPS survey

## Abstract

**Objective:**

The study aimed to evaluate the prevalence and correlates of sedentary behavior and combination of sedentary behavior and low physical activity among adults in Afghanistan in 2018.

**Methods:**

This was a national representative cross-sectional study. The study utilized the data from Afghanistan STEPS survey 2018, where 3,956 adults (ages between 18 and 69 years) were interviewed at community-dwelling level. Using the Global Physical Activity Questionnaire, we have calculated the sedentary behavior and physical inactivity. Logistic regression was applied to investigate factors associated high sedentary behavior and low physical activity.

**Results:**

Approximately half of the participants (49.8%) exhibited high levels of sedentary behavior, 40.3% low physical activity and 23.5% had both high sedentary behavior and low physical activity. Adjusted logistic regression analysis revealed that individuals who were employed (AOR: 0.34, 95% CI: 0.13–0.88) or self-employed (AOR: 0.60, 95% CI: 0.38–0.94) had significantly lower odds of both high SB and low physical activity than those whose work status was unpaid. Furthermore, older age (AOR: 1.75, 95% CI: 1.35–2.28), urban residence (AOR: 3.17, 95% CI: 1.72–6.05), having 4 or 5 adult household members (AOR: 1.77, 95% CI: 1.21–2.58) and being underweight (AOR: 1.78, 95% CI: 1.02–3.12) were found to be associated with high sedentary behavior. Moreover, factors such as female sex, having 4 or 5 or 6 or more adult household members, urban residence, overweight, and diabetes were positively associated, and male sex (AOR: 0.24, 95% CI: 0.12–0.51), being employed (AOR: 0.34, 95% CI: 0.13–0.88) or self-employed (AOR: 0.60, 95% CI: 0.38–0.94) were negatively associated with the occurrence of combination of high sedentary behavior and low physical activity.

**Conclusion:**

Half of the participants had high sedentary behavior, and one in four had both high sedentary behavior and low physical activity together. These findings emphasize the importance of targeted interventions aimed at reducing sedentary behavior and promoting physical activity, particularly among vulnerable populations such as females, individuals from lower socioeconomic background, urban residents, and those with chronic conditions. Addressing these factors can contribute to improving public health outcomes and reducing negative health impacts of sedentary behavior in Afghanistan.

## Introduction

Sedentary behavior (SB) has been described as “any waking behavior characterized by an energy expenditure of 1.5 metabolic equivalents (METS) or lower while sitting, reclining, or lying” (1). The significance of this problem extends beyond its mere prevalence; it encompasses a complex web of adverse health outcomes and socioeconomic implications that necessitates careful consideration. SB is not an isolated issue but a contributor to spectrum of health problems. Independent of individual’s physical activity (PA), SB has been identified as a critical factor in the development of several health conditions such a type 2 diabetes, cardio-metabolic risks, hypertension, high cholesterol (2–4). Its influence on this condition is profound leading to increased morbidity and mortality rates. In addition, there is an increasing negative impact of combination of SB and low PA on morbidity and mortality (5–7).

In studies in high-income countries, for example, among adults in Japan the prevalence of high SB (≥8 h/day) was 25.3% (8), and among adults across 28 European countries, the prevalence of high SB (>7.5 h/day) was 18.5% (9). Among adults in Australia, 8.9% had combination of high SB and low PA (10), and among adults in the USA the combination of high SB and low PA prevalence was 5.5% (11). In middle-income countries, for example, among adults in Armenia the prevalence of SB (≥8 h/day) was 13.2% (12), among adults in Bhutan, 8.2% (≥6 h/day) (13), among adults in South Africa 13.3% (≥8 h/day) (14), and among adults in six low-and middle-income countries (LMIC), the prevalence of high SB (≥8 h/day) was 8.3% (15).

Understanding the correlates of high SB and the combination of high SB and low PA is crucial for the development of appropriate interventions (8). Correlates of SB in high-income countries may include, for example in Japan, higher socioeconomic status, and higher body mass index (BMI) (≥25 kg/m^2^) (8), in Australia male sex, higher education, higher BMI and lower self-rated health (10). Correlates of SB in LMIC may include, for example, in Bhutan higher socioeconomic status, urban residence, low PA and diabetes (13), in South Africa, older age, cognitive impairment, hypertension and stroke (14), and in six LMIC, unemployment, tobacco use, low PA, functional disability, poorer mental and physical health status (15). Correlates for combination of high SB and low PA, for example among adults in Mexico, include sociodemographic factors including higher socioeconomic status, higher education, urban residence, and lower age (16).

In high-income countries, comprehensive research has highlighted the far-reaching consequences of SB and the pressing need for interventions. Some school-based programs in high income countries that encourage performing regular activity and avoids prolonged sitting, have demonstrated positive outcomes (17). Additionally, another initiative known as “Active School” program, implemented in Canada, which emphasize on high-quality physical education and actively encourages students to engage in 60 min or more of moderate- to vigorous PA, resulted in a significant impact on the health and well-being of participating students (18).

The extent to which these social and health correlates apply to adults in Afghanistan remains unclear, and requires further investigation. There is little information available on the epidemiology and associated factors of SB and combination of SB and low PA in LMIC, particularly in Afghanistan, which reduces our ability to design effective interventions (19). To address this research gap, we in this study aimed to evaluate the prevalence and correlates of SB and combination of SB and low PA among adults in Afghanistan. By clarifying the problem’s magnitude and underpinning its consequences, this research seeks to inform policy makers, public health practitioners, and the global health community about the urgent need for targeted interventions. In a country striving to rebuild its health systems and improve the well-being of its citizens, this study will serve as empirical evidence which will guide the development of evidence-based strategies aiming to reduce sedentary behavior and promoting PA, which ultimately will enhance the quality of life and well-being of the population.

## Methods

### Sample and procedure

**Figure 1:**
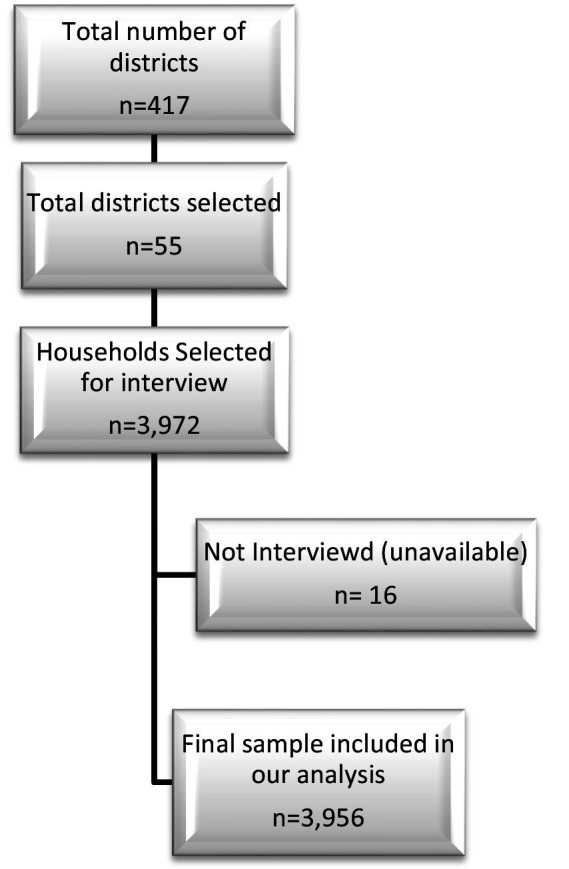
Flowchart of the sample selection.

This analysis used secondary data from a national cross-sectional household survey in Afghanistan in 2018 (20). By using a multistage cluster approach, a nationally representative sample of individuals aged 18–69 years was generated (21). The primary sampling units were 55 districts randomly selected from 417 districts, followed by selection of households from these districts proportionate to the size of district (see Figure 1). One person from each household was randomly selected (21). The STEPS recommended 3 age groups per gender of 18–29, 30–44, and 45–69 years were used to calculate the sample size for the 6 different strata of populations. Using a confidence level of 95%, a margin of error of 5%, 0.5p, and 0.5q, the resulting sample size was 384. With a design effect of 1.5 and a non-response rate of 15%, the sample size was adjusted to 662 for each strata of the age-sex group. The adjusted sample size was multiplied by six gender groups (662*6) to get the final sample size of 3,972 households. In the end, 3,972 households (male and female) were selected for data collection in 55 randomly selected districts of Afghanistan. The dataset lacked 16 households, so the final sample size included in the analysis included 3,956 households (21).

Inclusion criteria were household permanent residents aged 18–69 (50% male and 50% females) and willingness to participate in the study (21). To insure the cultural sensitivity, interviews were conducted by trained interviewers of the same gender, with male interviewers for males participants and female interviewers for female participants (21). In the sedentary behaviors measurement, there were 24 (0.6%) missing observations and for PA, there were 36 (0.5%) missing observations. Calculating the difference in characteristics between the excluded subjects and the included subjects, we could not find any significant differences (*p* > 0.05). Ethical approval for the original survey was obtained from the “Ministry of Public Health Ethics Board” in Afghanistan, and participants provided written informed consents.

Data collection followed the “WHO STEPS methodology: step 1 included administrating a structured questionnaire (sociodemographics, medical history, medication use, and health risk behaviors), step 2 entailed measuring blood pressure and anthropometric indicators, and step 3 encompassed conducting biochemical tests (blood glucose and blood lipids assessments)” (21).

### Measures

#### Outcome variables

SB was assessed with one item from the “Global Physical Activity Questionnaire (GPAQ)” (22), as follows:

“The following question is about sitting or reclining at work, at home, getting to and from places, or with friends including time spent sitting at a desk, sitting with friends, traveling in car, bus, train, reading, playing cards or watching television, but do not include time spent sleeping. How much time do you usually spend sitting or reclining on a typical day?” (Hours/min).

High SB was classified as 8 or more hours per day, following a previous classification for all-time mortality risk (23). Data truncation was applied when sedentary duration was 960 min/day (16 h) or more (22).

*Physical activity* levels were classified into low, moderate, and high PA (<600, 600–1,500, and > 1,500 MET-minutes/week, respectively) according to the GPAQ guidelines (22). Data truncation was applied when the activity reported within any domain or intensity was 960 min/day (16 h) or more (22). Occurrence of high SB and low PA together was defined as combination of SB and low PA.

Social and demographic informations included, age, sex, education, number of adult household members (as a proxy for socioeconomic status) (24), and residence status. Past 12-month work status was grouped into 1 = employee (government employee, or non-government employee), 2 = self-employed, and 3 = unpaid (student, unemployed, homemaker, non-paid, or retired) (21).

Behavioral covariates included current tobacco use, and daily servings of vegetable and fruit intake.

Biological variables included BMI, blood pressure, blood sugar, hypercholesterolemia, and heart attack. BMI level was classified as “<18.5 kg/m^2^ underweight, 18.5–24.9 kg/m^2^ normal weight, 25–29.9 kg/m^2^ overweight and ≥30 kg/m^2^ obesity” (25). *Hypertension* was defined as “systolic blood pressure (BP) ≥140 mmHg and/or diastolic BP ≥90 mm Hg or where the participant is currently on antihypertensive medication” (26). *Diabetes* was defined as “fasting plasma glucose levels ≥7.0 mmol/L (126 mg/dL); or using insulin or oral hypoglycemic drugs; or having a history of diagnosis of diabetes” (27). Raised total cholesterol (TC) was defined as “fasting TC ≥5.0 mmol/L or currently on medication for raised cholesterol” (27). History of heart attack or stroke was assessed from the question, “Have you ever had a heart attack or chest pain from heart disease (angina) or a stroke (cerebrovascular accident or incident)?” (21).

### Data analysis

Analysis weights were calculated by taking the inverse of the probability of selection of each participant adjusted for differences in the age-sex composition of the sample population as compared to the target population (21). Descriptive statistics were used to provide the distribution of sociodemographic and health information of the sample. Unadjusted and adjusted logistic regression was applied to investigate associated factors (sociodemographic and health variables) of high SB and combination of high SB and low PA. Variables significant in univariate analyses were subsequently included in the multivariable logistic regression. Taylor linearization methods were applied in statistical procedures accounting for sample weight and multi-stage sampling. Only complete cases were included (<0.5% missing on outcome variables SB and PA) in the analysis and significance was established at *p* < 0.05. Statistical procedures were done using “Stata software version 15.1 (Stata Corporation, College Station, TX, United States),” and considering the complex study approach of multi-stage sampling and weighting of the data.

## Results

### Sample characteristics

The sample consisted of 3,956 adults (18–69 years), with a median age of 35 years (interquartile range 24–60), 51.9% of the participants were men. Majority (61.1%) had no formal education, 42.3% were living with six or more adult household members, and 57.8% lived in urban areas. Approximately half of the participants (49.8%) had high SB, 40.3% low PA, and 23.5% had both high SB and low PA. Further sociodemographic details and information about health variables are shown in Table 1.

**Table 1:** Sample and sedentary behavior (SB) and low physical activity (PA) characteristics among adults in Afghanistan, 2018.

Variable	Sample	SB	Low PA	SB and low PA
	N^a^ (%)^b^	%^b^	%^b^	%^b^
All	3,956	49.8	40.3	23.5
Age in years				
18–29	1879 (47.9)	46.7	40.0	21.9
30–69	2046 (52.1)	54.0	40.7	25.6
Sex				
Female	1930 (48.1)	58.9	62.9	39.2
Male	2022 (51.9)	41.5	19.5	9.0
Education				
None	2,225 (61.1)	53.0	48.6	30.2
≤Primary	681 (15.8)	48.9	37.8	19.1
≥Secondary	1,047 (23.1)	42.2	20.1	8.8
Adult household members				
1–3	1,412 (23.8)	40.3	33.2	15.7
4–5	1,286 (34.0)	55.0	37.8	23.0
≥6	1,255 (42.3)	51.1	46.3	28.3
Work status				
Unpaid	2,134 (55.9)	55.3	55.6	35.2
Employee	346 (8.4)	41.9	20.6	9.4
Self-employed	1,457 (35.7)	48.0	16.0	6.4
Residence				
Rural	1877 (42.2)	33.6	31.5	14.3
Urban	2078 (57.8)	61.7	46.7	30.2
Body mass index				
Normal	1774 (49.5)	42.8	32.0	16.7
Underweight	264 (7.8)	56.6	37.3	21.4
Overweight	1,071 (25.5)	51.0	40.6	24.6
Obesity	636 (17.2)	54.7	52.8	29.6
Current tobacco use	870 (26.2)	49.0	27.2	15.3
Daily servings of fruit/vegetables intake				
≤1	2,523 (59.8)	48.6	43.0	26.0
2	925 (28.9)	54.4	35.1	20.6
≥3	508 (11.3)	44.6	39.3	17.6
Hypertension	1,193 (29.2)	51.6	47.1	28.4
Type 2 diabetes	408 (9.2)	59.7	59.1	39.6
Raised cholesterol	707 (18.0)	56.4	47.1	32.4
Heart disease or stroke	293 (8.8)	40.7	27.3	14.2

^a^Unweighted, ^b^weighted.

### Associations with high sedentary behavior

In univariable analyses, older age, having 4–5 adult household members, urban residence, overweight, obesity, and type 2 diabetes were positively associated with high SB, while male sex, being self-employed and high PA were negatively associated with high SB. In the multivariable analysis, older age (30–69 years) (AOR: 1.75, 95% CI: 1.35–2.28), having 4–5 adult household members (AOR: 1.77, 95% CI: 1.21–2.58), urban residence (AOR: 3.23, 95% CI: 1.72–6.05) and being underweight (AOR: 1.78, 95% CI: 1.02–3.12) were significantly positively associated with high SB, and being male (AOR: 0.50, 95% CI: 0.29–0.91) was inversely associated with high SB (see Table 2).

**Table 2:** Association of sociodemographic and health variables with high sedentary behavior among adults in Afghanistan, 2018.

Variable	CrOR (95% CI)	*p*	AOR (95% CI)	*p*
Sociodemographic variables				
Age in years				
18–29	1 (Reference)		1 (Reference)	
30–69	1.34 (1.02, 1.76)	0.036	1.75 (1.35, 2.28)	<0.001
Sex				
Female	1 (Reference)		1 (Reference)	
Male	0.50 (0.27, 0.92)	0.025	0.50 (0.29, 0.91)	0.023
Education				
None	1 (Reference)		1 (Reference)	
≤Primary	0.85 (0.59, 1.22)	0.375	0.98 (0.71, 1.35)	0.890
≥Secondary	0.65 (0.38, 1.12)	0.119	0.92 (0.62, 1.37)	0.680
Adult household members				
1–3	1 (Reference)		1 (Reference)	
4–5	1.81 (1.18, 2.79)	0.007	1.77 (1.21, 2.58)	0.003
≥6	1.55 (0.91, 2.65)	0.131	1.27 (0.80, 2.00)	0.312
Work status				
Unpaid	1 (Reference)		1 (Reference)	
Employee	0.75 (0.34, 1.65)	0.465	1.56 (0.84, 2.90)	0.163
Self-employed	0.58 (0.35, 0.98)	0.040	1.31 (0.84, 2.03)	0.227
Residence				
Rural	1 (Reference)		1 (Reference)	
Urban	3.17 (1.92, 5.24)	<0.001	3.23 (1.72, 6.05)	<0.001
Health variables				
Body mass index				
Normal	1 (Reference)		1 (Reference)	
Underweight	1.74 (0.99, 3.04)	0.052	1.78 (1.02, 3.12)	0.042
Overweight	1.39 (1.04, 1.85)	0.026	1.24 (0.90, 1.70)	0.192
Obesity	1.61 (1.18, 2.20)	0.003	1.26 (0.89, 1.80)	0.197
Physical activity				
Low	1 (Reference)		1 (Reference)	
Moderate	0.68 (0.40, 1.18)	0.242	1.28 (0.73, 2.27)	0.388
High	0.53 (0.30, 0.94)	0.043	0.86 (0.54, 1.36)	0.520
Current tobacco use	0.95 (0.59, 1.84)	0.844	1.35 (0.99, 1.85)	0.054
Daily servings of fruit/vegetables intake				
≤1	1 (Reference)		1 (Reference)	
2	1.26 (0.83, 1.91)	0.271	1.29 (0.89, 1.87)	0.177
≥3	0.85 (0.51, 1.40)	0.521	0.80 (0.47, 1.35)	0.401
Hypertension	1.09 (0.86, 1.39)	0.482	1.01 (0.77, 1.33)	0.915
Type 2 diabetes	1.62 (1.06, 2.49)	0.026	1.19 (0.78, 1.81)	0.423
Raised cholesterol	1.44 (0.95, 2.18)	0.082	1.08 (0.75, 1.55)	0.683
Heart disease or stroke	0.67 (0.34, 1.32)	0.242	0.67 (0.33, 1.36)	0.268

CrOR, Crude Odds Ratio; AOR, Adjusted Odds Ratio.

### Associations with low physical activity

In univariable analyses, having 6 or more adult household members, urban residence, overweight, obesity, hypertension and type 2 diabetes were positively associated with low PA, while male sex, higher education, being employed or self-employed, current tobacco use and having a history of heart attack or stroke were negatively associated with low PA. In the multivariable analysis, having 6 or more adult household members (AOR: 1.88, 95% CI: 1.24–2.84), urban residence (AOR: 2.12, 95% CI: 1.34–3.38), and being overweight (AOR: 1.36, 95% CI: 1.04–1.78) were significantly positively associated with low PA, and being male (AOR: 0.22, 95% CI: 0.10–0.50), having secondary or higher education (AOR: 0.58, 95% CI: 0.36–0.93), and being employed (AOR: 0.34, 95% CI: 0.16–0.71) were inversely associated with low PA (see Table 3).

**Table 3:** Association of sociodemographic and health variables with low physical activity among adults in Afghanistan, 2018.

Variable	CrOR (95% CI)	*p*	AOR (95% CI)	*p*
Sociodemographic variables				
Age in years				
18–29	1 (Reference)		1 (Reference)	
30–69	1.03 (0.82, 1.31)	0.790	1.01 (0.70, 1.46)	0.958
Sex				
Female	1 (Reference)		1 (Reference)	
Male	0.24 (0.08, 0.26)	<0.001	0.22 (0.10, 0.50)	<0.001
Education				
None	1 (Reference)		1 (Reference)	
≤Primary	0.64 (0.43, 0.96)	0.031	0.92 (0.57, 1.50)	0.745
≥Secondary	0.27 (0.18, 0.40)	<0.001	0.58 (0.36, 0.93)	0.023
Adult household members				
1–3	1 (Reference)		1 (Reference)	
4–5	1.22 (0.81, 1.86)	0.342	1.27 (0.91, 1.78)	0.164
≥6	1.73 (1.12, 2.67)	0.013	1.88 (1.24, 2.84)	0.003
Work status				
Unpaid	1 (Reference)		1 (Reference)	
Employee	0.15 (0.08, 0.29)	<0.001	0.34 (0.16, 0.71)	0.004
Self-employed	0.20 (0.12, 0.34)	<0.001	0.60 (0.32, 1.10)	0.100
Residence				
Rural	1 (Reference)		1 (Reference)	
Urban	1.91 (1.04, 3.50)	0.036	2.12 (1.34, 3.38)	0.002
Health variables				
Body mass index				
Normal	1 (Reference)		1 (Reference)	
Underweight	1.26 (0.75, 2.14)	0.378	1.09 (0.65, 1.85)	0.741
Overweight	1.45 (1.15, 1.84)	0.002	1.36 (1.04, 1.78)	0.023
Obesity	2.38 (1.66, 3.40)	<0.001	1.50 (0.95, 2.35)	0.079
High sedentary behavior	1.70 (0.98, 2.93)	0.059	1.02 (0.64, 1.62)	0.945
Current tobacco use	0.46 (0.26, 0.78)	0.004	1.22 (0.73, 2.04)	0.451
Daily servings of fruit/vegetables intake				
≤1	1 (Reference)	0.151	1 (Reference)	0.234
2	0.75 (0.50, 1.11)	0.635	0.79 (0.53, 1.17)	0.381
≥3	0.88 (0.51, 1.51)		0.83 (0.55, 1.26)	
Hypertension	1.91 (1.04, 3.50)	0.003	1.25 (0.95, 1.64)	0.114
Type 2 diabetes	2.67 (1.58, 4.52)	<0.001	1.83 (0.98, 3.42)	0.057
Raised cholesterol	1.49 (0.98, 2.25)	0.062	0.81 (0.54, 1.22)	0.313
Heart disease or stroke	0.53 (0.30, 0.92)	0.025	0.70 (0.36, 1.35)	0.114

CrOR, Crude Odds Ratio; AOR, Adjusted Odds Ratio.

### Associations with combination of high sedentary behavior and low physical activity

In univariable analyses, having six or more adult household members, urban residence, overweight, obesity, hypertension, type 2 diabetes, and raised cholesterol were positively associated with combination of SB and low PA, while male sex, higher education, being employed or self-employed, current tobacco use and having heart disease or stroke were negatively associated. In multivariable analysis, compared to participants whose work status was unpaid, the odds of combination of high SB and low PA was significantly lower in employees (AOR: 0.34, 95% CI: 0.13–0.88), and those self-employed (AOR: 0.60, 95% CI: 0.38–0.94). Furthermore, the male gender (AOR: 0.24, 95% CI: 0.12–0.51) and consumption of 3 or more servings of fruit and vegetables a day (AOR: 0.50, 95% CI: 0.28–0.88) exhibited negative correlations, whereas, having 6 or more adult household members (AOR: 2.39, 95% CI: 1.42–4.04), residing in urban areas (AOR: 2.77, 95% CI: 1.60–4.81), being overweight (AOR: 1.45, 95% CI: 1.05–1.99), and having type 2 diabetes (AOR: 2.02, 95% CI: 1.20–3.43) were positively associated with the co-occurrence of high SB and low PA (see Table 4).

**Table 4:** Association of sociodemographic and health variables with combination of high sedentary behavior and low physical activity among adults in Afghanistan, 2018.

Variable	CrOR (95% CI)	*p*	AOR (95% CI)	*p*
Sociodemographic variables				
Age in years				
18–29	1 (Reference)		1 (Reference)	
30–69	1.23 (0.91, 1.64)	0.175	1.42 (0.99, 2.03)	0.054
Sex				
Female	1 (Reference)		1 (Reference)	
Male	0.15 (0.09, 0.28)	<0.001	0.24 (0.12, 0.51)	<0.001
Education				
None	1 (Reference)		1 (Reference)	
≤Primary	0.54 (0.34, 0.86)	0.013	0.80 (0.45, 1.42)	0.440
≥Secondary	0.22 (0.13, 0.38)	<0.001	0.72 (0.40, 1.30)	0.274
Adult household members				
1–3	1 (Reference)		1 (Reference)	
4–5	1.60 (0.99, 2.61)	0.057	1.78 (1.10, 2.88)	0.018
≥6	2.12 (1.28, 2.50)	0.004	2.39 (1.42, 4.04)	<0.001
Work status				
Unpaid	1 (Reference)		1 (Reference)	
Employee	0.13 (0.06, 0.27)	<0.001	0.34 (0.13, 0.88)	0.026
Self-employed	0.19 (0.11, 0.33)	<0.001	0.60 (0.38, 0.94)	0.025
Residence				
Rural	1 (Reference)		1 (Reference)	
Urban	2.60 (1.39, 4.87)	<0.001	2.77 (1.60, 4.81)	<0.001
Health variables				
Body mass index				
Normal	1 (Reference)		1 (Reference)	
Underweight	1.36 (0.73, 2.55)	0.327	1.40 (0.74, 2.67)	0.304
Overweight	1.63 (1.22, 2.17)	<0.001	1.45 (1.05, 1.99)	0.023
Obesity	2.10 (1.36, 3.25)	<0.001	1.06 (0.65, 1.74)	0.813
Current tobacco use	0.50 (0.28, 0.91)	0.024	1.36 (0.69, 2.67)	0.377
Daily servings of fruit/vegetables intake				
≤1	1 (Reference)		1 (Reference)	0.593
2	0.74 (0.47, 1.16)	0.190	0.88 (0.56, 1.39)	0.016
≥3	0.61 (0.37, 1.01)	0.054	0.50 (0.28, 0.88)	
Hypertension	1.46 (1.05, 2.13)	0.026	1.35 (0.96, 1.90)	0.085
Type 2 diabetes	2.51 (1.49, 4.25)	<0.001	1.88 (1.10, 2.88)	0.021
Raised cholesterol	1.90 (1.24, 2.89)	0.003	1.08 (0.74, 1.58)	0.672
Heart disease or stroke	0.51 (0.27, 0.99)	0.045	0.63 (0.33, 1.22)	0.170

CrOR, Crude Odds Ratio; AOR, Adjusted Odds Ratio.

## Discussion

We found that the proportion of high SB (49.2%) in Afghanistan was higher compared to some national community-based surveys using similar self-reported measures in low resourced countries, such as in Armenia (13.2%) (12), in South Africa (13.3%) (14), and in Bhutan (8.2%) (13, 15), and community-based studies in some high-income countries, for example, in Japan (25.3%) (8), and 28 countries in Europe (18.5%) (9). In small cross-sectional population-based studies among adults in urban Afghanistan, e.g., in Kandahar city in 2019 also a high rate of daily sitting time (average 10.4 h, compared to 6.8 h in this study) has been reported (28) and in Jalalabad city the prevalence of sitting 3 or more hours a day was 35.1% (29). The prevalence of low PA (40.3%) in this study was higher than in a cross-sectional study among hospital patients in Kandahar city (27%) (30). Regarding the prevalence of the combination of high SB and low PA (23.5%) in Afghanistan, the observed rates were found to be significantly higher compared to previous national community-based studies conducted in other regions, such as in Australia (8.9%) (10), and the USA during the 2017/2018 period (5.5%) (11). The elevated prevalence of SB and the co-occurrence of high SB and low PA in Afghanistan can be attributed to various factors such as urbanization, changes in occupational patterns involving more SB, and an increase in less active transportation in both urban and rural areas (19). Among the obstacles to participation in PA in Afghanistan are the lack of time, being too tired, a lack of confidence in participating in certain types of PA, the type of clothing often worn during exercise, the lack of single-sex facilities, the inability to participate in PA with men and the need to be completely covered outside the house (31). Furthermore, recent government actions have intensified the issue, with bans on women’s access to work, education and specifically public parks. These restrictions which further exacerbates sedentary behavior necessitates immediate attention and the implementation of transformative interventions to promote PA.

It is crucial to implement interventions that specifically target SB and the combined occurrence of SB and low PA. The interventions may include public awareness campaigns, normalizing PA, national PA campaigns, national mass participation events on PA, improving access to sport and other PA facilities, including single-sex facilities, brief intervention on PA in primary care, and apart from already promoting PA in public open spaces, PA can be promoted in workplaces, childcare, school and university setting, through community sports, through walking and cycling, for older adults and for people with disability (19, 31–33).

Our study revealed several factors that were associated with increased odds of high SB and co-occurrence of high SB and low PA. These factors included non-work status, older age, urban residence and being underweight which were all positively associated with high SB. Furthermore, non-work status, female sex, lower socioeconomic status, urban residence, being overweight, lower fruit and vegetable intake and having diabetes were associated with the odds of having both high SB and low PA. Consistent with previous studies (8, 12, 13, 16, 34–37) we found that older age, female sex, and urban residence were positively associated with high SB and/or combination of high SB and low PA. The higher prevalence of SB, low PA and combination of high SB and low PA among women than men, “could be related to cultural issues in the Afghan context such as access to physical exercise facilities and restriction of female movement outside the home” (19). Cities in Afghanistan may be exposed to increased traffic and crime and increased use of motorized transport, leading to increased SB (15). These results support the implementation of interventions aimed at reducing SB among women residing in urban areas of Afghanistan (15). The finding that lower fruit and vegetable intake was associated with the odds of having both high SB and low PA may be explained by clustering of risk factors of non-communicable diseases (29).

While some research (10, 13, 16, 35) found a positive association between higher socioeconomic status and high SB, we found no consistent significant association between higher socioeconomic status (higher education, lower number of adult household members), and a negative association between higher socioeconomic status (lower number of adult household members) and high SB and/or combination of high SB and low PA, and those with higher education had lower odds of low PA. Furthermore, compared to participants who had an unpaid work status, the odds of combination of high SB and low PA was significantly lower in those who were employed or self-employed. People who are employed or self-employed, have better education and have better economic status may be more aware of the importance of PA and have more opportunities to engage in PA (15).

In terms of health-related factors, our findings were consistent with previous research (10, 13–15), indicating that overweight, and diabetes were associated with combination of high SB and low PA. Individuals who are overweight may experience a decline in mobility that reduces their energy expenditure, leading to weight gain. This weigh gain, in turn, further reduces mobility and promotes sedentary lifestyle (38, 39). It is also plausible that high levels of SB and low PA contribute to chronic conditions (15). Our findings, however, suggest that SB interventions should consider persons with chronic conditions, such as overweight, and diabetes (15).

In unadjusted analysis, high PA, current tobacco use, and history of heart disease or stroke were negatively associated with high SB and/or combination of SB and low PA. Conversely, raised cholesterol levels and hypertension were positively associated with these outcomes. Previous research (13, 15) has also shown an association between low PA and high SB. Our study found that current tobacco use was marginally associated with high SB, which is in consistence with previous studies (10, 13, 15). It is worth noting that SB can contribute to decreased cardiorespiratory fitness and an increased risk of hypertension, coronary heart disease, and stroke (40). In a previous study a high prevalence of SB was found in stroke survivors (41). Engaging stroke survivors in PA may be difficult to achieve, but it would be important to develop adapted possible strategies of PA in this group (42).

### Study limitations

The cross-sectional nature of our survey hinders us in drawing causal conclusions. For example, the direction of the association between overweight and combination of high SB and low PA could be bi-directional, meaning that combination of high SB and low PA could lead to overweight and overweight could lead to combination of high SB and low PA. Additionally certain data in our study relied on self-report measures, including SB and PA, which may have introduced response bias potentially leading to underestimation of SB time (43). Furthermore, we only assessed overall SB, instead of assessing separate SB domains, such as leisure time, transport and work.

## Conclusion

Half of adults in Afghanistan had high SB, two in five low PA and one in four had both high SB and low PA. Older age, female sex, urban residence, having 4 or 5 adult household members, and being underweight increased the odds of high SB. Furthermore, non-work status, female sex, lower socioeconomic status, urban residence, overweight, lower intake of fruit and vegetables, and diabetes increased the odds of combination of high SB and low PA. Interventions aimed at reducing SB and promoting PA should target specific subgroups such as females, older individuals, urban residents, those with chronic conditions (underweight, overweight, and diabetes) and those with lower socioeconomic status.

## Data availability statement

Publicly available datasets were analyzed in this study. This data can be found here: WHO NCD Microdata Repository (URL: https://extranet.who.int/ncdsmicrodata/index.php/catalog).

## Ethics statement

The studies involving humans were approved by Ethics approval for the STEPS survey was obtained from the “Ministry of Public Health Ethics Board” and participants provided informed consent. The studies were conducted in accordance with the local legislation and institutional requirements. The participants provided their written informed consent to participate in this study.

## Author contributions

SP, AN, and KP conceived and designed the research, performed statistical analysis, drafted the manuscript, and made critical revision of the manuscript for key intellectual content. All authors fulfil the criteria for authorship, read and approved the final version of the manuscript, and agreed to authorship and order of authorship for this manuscript.

## Conflict of interest

The authors declare that the research was conducted in the absence of any commercial or financial relationships that could be construed as a potential conflict of interest.

## Publisher’s note

All claims expressed in this article are solely those of the authors and do not necessarily represent those of their affiliated organizations, or those of the publisher, the editors and the reviewers. Any product that may be evaluated in this article, or claim that may be made by its manufacturer, is not guaranteed or endorsed by the publisher.
